
JOURNAL INFORMATION
==============================
NLM Title Abbreviation: ScientificWorldJournal
Journal ID: ScientificWorldJournal
NLM Journal ID: 101131163
Journal ID: TSWJ

The Scientific World Journal

ISSN: 2356-6140
EISSN: 1537-744X
Publisher: Wiley

ARTICLE INFORMATION
==============================
PMCID: PMC6009561
PMID: 29973791
DOI: 10.1100/tsw.2002.17
Article ID: 586381
Article version: 1
Subjects: Short Report

Automated Protein Purification for Structural Studies

Andersson Hans O.
Eklund Par
Galin Markus
Hedqvist Lotta
Liljedahl Pia *
Markeland Christine
Pless Thomas
Sigrell Jill A.
Torstenson Karin
Amersham Biosciences, Björkgatan 30, SE-751 84 Uppsala, Sweden
*Pia Liljedahl: jill.sigrell@eu.amershambiosciences.com
Publication date: 2002
Electronic publication date: 2002 Jan 29
Volume: 2
First page: 33
Last page: 34
Copyright: Copyright © 2002 Hans O. Andersson et al.
Copyright year: 2002
License: This is an open access article distributed under the Creative Commons Attribution License, which permits unrestricted use, distribution, and reproduction in any medium, provided the original work is properly cited.
License URL: https://creativecommons.org/licenses/by/3.0/

==============================
Advances in Gene Technology: The Genome and Beyond –
Structural Biology for Medicine (Proceedings of the 2002 Miami
*Nature Biotechnology* Winter Symposium)
*TheScientificWorld* 2002, 2(S2), 33–34
ISSN 1532-2246; DOI 10.1100/tsw.2002.17

### AUTOMATED PROTEIN PURIFICATION FOR STRUCTURAL STUDIES

Hans O. Andersson, Pär Eklund, Markus Galin, Lotta Hedqvist, Pia Liljedahl, Christine Markeland, Thomas Pless, Jill A. Sigrell*, and Karin Torstenson
Amersham Biosciences, Björkgatan 30, SE-751 84 Uppsala, Sweden
*jill.sigrell@eu.amershambiosciences.com

**INTRODUCTION.** Structural genomics, also known as structural proteomics, aims to resolve protein functions on a large scale[1]. To determine structures using either X-ray crystallography or NMR, fairly large amounts (>10 mg) of target proteins (which are pure, homogenous, and concentrated) are usually required. A robust and easy-to-use protein purification scheme that can automatically produce numerous proteins in a desired form would increase the throughput of structure determination[2]. Using a new strategy and optimized methods, a newly configured ÄKTAexplorer™ (ÄKTA is a trademark of the Amersham Biosciences group. Amersham and Amersham Biosciences are trademarks of Amersham Plc. © Amersham Biosciences AB 2001 – All rights reserved.) 100 can perform automated two-step purification of up to six different proteins serially using two different purification schemes.

**METHODS.** Ten different soluble and well-expressed recombinant proteins tagged with either $(His)_6$ or Glutathione S-transferase (GST) were produced in *Escherichia coli*. These proteins, with varying molecular weights (20–70 kD) and pIs (5.1–9.2), were purified using two different purification schemes: (1) affinity chromatography followed by gel filtration and (2) affinity chromatography followed by an intermediate desalting step prior to ion exchange chromatography.

Each sample was first applied and washed on an affinity column matching the affinity tag. By using one column for each protein, the different proteins could be captured one by one in a quick serial mode, thereby minimizing contact with proteases present in the homogenized *E. coli* extracts. Automation of the chosen purification schemes was achieved by using the added sample loops as reinjection devices in combination with step-elution. The eluted protein from one column (affinity or desalting) was directed to a sample loop before being injected to the next column.

**RESULTS AND DISCUSSION.** Production of six different pure proteins could be achieved within 12 h using scheme A and four proteins within 4 h using scheme B. Purification of all proteins using any of the purification schemes resulted in up-to-50 mg of 90–99% pure protein. For most proteins, ion exchange chromatography removed impurities to a higher degree than gel filtration. However, the ability of gel filtration to remove aggregates often makes it a valuable final step in preparing homogenous protein samples for structural studies. With this new configuration, the researcher saves time compared to the same chromatography steps performed in a nonautomated manner.

**ACKNOWLEDGEMENTS.** Amersham Biosciences is grateful to Structural GenomiX for providing several of the tested His-tagged proteins.

**REFERENCES**

1. Structural genomics supplement (2000) Nat. Struct. Biol. 7, 927–994.
2. Lesley, S.A. (2001) Prot. Express. Purif. 22, 159–164.
